# Rapamycin modulates tissue aging and lifespan independently of the gut microbiota in *Drosophila*

**DOI:** 10.1038/s41598-019-44106-5

**Published:** 2019-05-24

**Authors:** Joseph M. Schinaman, Anil Rana, William W. Ja, Rebecca I. Clark, David W. Walker

**Affiliations:** 1 Department of Integrative Biology and Physiology, University of California, Los Angeles, 90095 Los Angeles, California USA; 2 Department of Neuroscience, The Scripps Research Institute, 33458 Jupiter, FL USA; 3 Center on Aging, The Scripps Research Institute, 33458 Jupiter, FL USA; 4 Department of Biosciences, Durham University, DH1 3LE Durham, UK; 5 Molecular Biology Institute, University of California, Los Angeles, 90095 Los Angeles, California USA

**Keywords:** Protein aggregation, Ageing

## Abstract

The FDA approved drug rapamycin can prolong lifespan in diverse species and delay the onset of age-related disease in mammals. However, a number of fundamental questions remain unanswered regarding the mechanisms by which rapamycin modulates age-related pathophysiology and lifespan. Alterations in the gut microbiota can impact host physiology, metabolism and lifespan. While recent studies have shown that rapamycin treatment alters the gut microbiota in aged animals, the causal relationships between rapamycin treatment, microbiota dynamics and aging are not known. Here, using *Drosophila* as a model organism, we show that rapamycin-mediated alterations in microbiota dynamics in aged flies are associated with improved markers of intestinal and muscle aging. Critically, however, we show that the beneficial effects of rapamycin treatment on tissue aging and lifespan are not dependent upon the microbiota. Indeed, germ-free flies show delayed onset of intestinal barrier dysfunction, improved proteostasis in aged muscles and a significant lifespan extension upon rapamycin treatment. In contrast, genetic inhibition of autophagy impairs the ability of rapamycin to mediate improved gut health and proteostasis during aging. Our results indicate that rapamycin-mediated modulation of the microbiota in aged animals is not causally required to slow tissue and organismal aging.

## Introduction

In recent years, there has been a growing appreciation of the potential impact of developing effective interventions to slow aging and prolong healthy lifespan^1,2^. A leading target for such interventions is the nutrient-sensing mechanistic Target of Rapamycin (mTOR) pathway^3,4^. The mTOR protein kinase is found in two discrete complexes, mTOR complex 1 (mTORC1) and mTORC2, each of which contains distinct protein components and phosphorylates different substrates^5^. Rapamycin, a small molecule that acutely inhibits mTORC1, can extend lifespan and delay the onset of age-related diseases in model organisms including flies and mice^3,4,6–12^. Hence, there is significant interest in elucidating the underlying mechanisms of rapamycin-mediated longevity. The accumulation of damaged and misfolded proteins due to a loss of protein homeostasis (proteostasis) is a shared hallmark of aging and numerous age-onset diseases^13–15^. Inhibiting mTORC1 activity can induce autophagy^5,16^, a cellular recycling process that can eliminate damaged proteins and organelles^17,18^. However, the interplay between rapamycin treatment, autophagy, proteostasis and healthy aging remains incompletely understood.

The diverse collection of commensal and symbiotic microbes (microbiota) that live in close association with many animal species can profoundly influence many aspects of host physiology, including nutrient metabolism and immune function. Indeed, there is an emerging understanding that the microbiota can influence host aging and lifespan determination^19–21^. Age-related alterations in microbiota composition have been reported in flies, fish, mice and humans^22–27^. In both flies and mice, age-onset microbial dysbiosis contributes to intestinal barrier dysfunction, a conserved pathophysiological hallmark of aging^23,28^. Remarkably, recolonizing the gut of middle-aged fish with bacteria from young donors prolongs lifespan and delays behavioral decline^27^. In addition, recent work has shown that microbial genetic composition and metabolites can positively impact host longevity^29,30^. Together, these findings raise the intriguing possibility that existing interventions that promote longevity do so via an interplay with the microbiota. Consistent with this idea, the lifespan-extending effects of metformin in *C*. *elegans* are eliminated when worms are cultured axenically (germ-free)^31^. However, there is a scarcity of data addressing the question of whether the microbiota plays an important role in mediating additional prolongevity interventions.

Rapamycin treatment has been reported to alter microbiota dynamics in both aged flies^32^ and mice^33^. More specifically, rapamycin treatment was reported to reduce bacterial load in aged fly guts, however, it was not determined whether the composition of the microbiota was altered^32^. Moreover, the question of whether alterations of the microbiota play a causal role in the beneficial effects associated with rapamycin treatment remains to be answered. Here, we show that rapamycin treatment leads to reduced levels of Alphaproteobacteria, a taxon that has previously been linked to health decline and mortality in aged flies^23^. Consistent with a previous report^32^, we find that rapamycin treatment maintains intestinal barrier function during aging. Importantly, we show that functional autophagy is required for the rapamycin-mediated improvement in gut function in aged flies. In addition, we show that rapamycin treatment improves proteostasis in aged muscles in an autophagy-dependent manner. Critically, however, we find that eliminating the microbiota does not prevent the anti-aging effects of rapamycin at either the tissue or organismal level. Moreover, we show that germ-free flies show improved lifespan and proteostasis upon rapamycin treatment compared to microbe-bearing control animals. Our findings demonstrate that rapamycin, provided in the food, can act directly on the host organism to maintain tissue homeostasis during aging in an autophagy-dependent fashion.

## Results

### Rapamycin treatment improves gut health and modulates microbiota composition during aging

**Figure 1:**
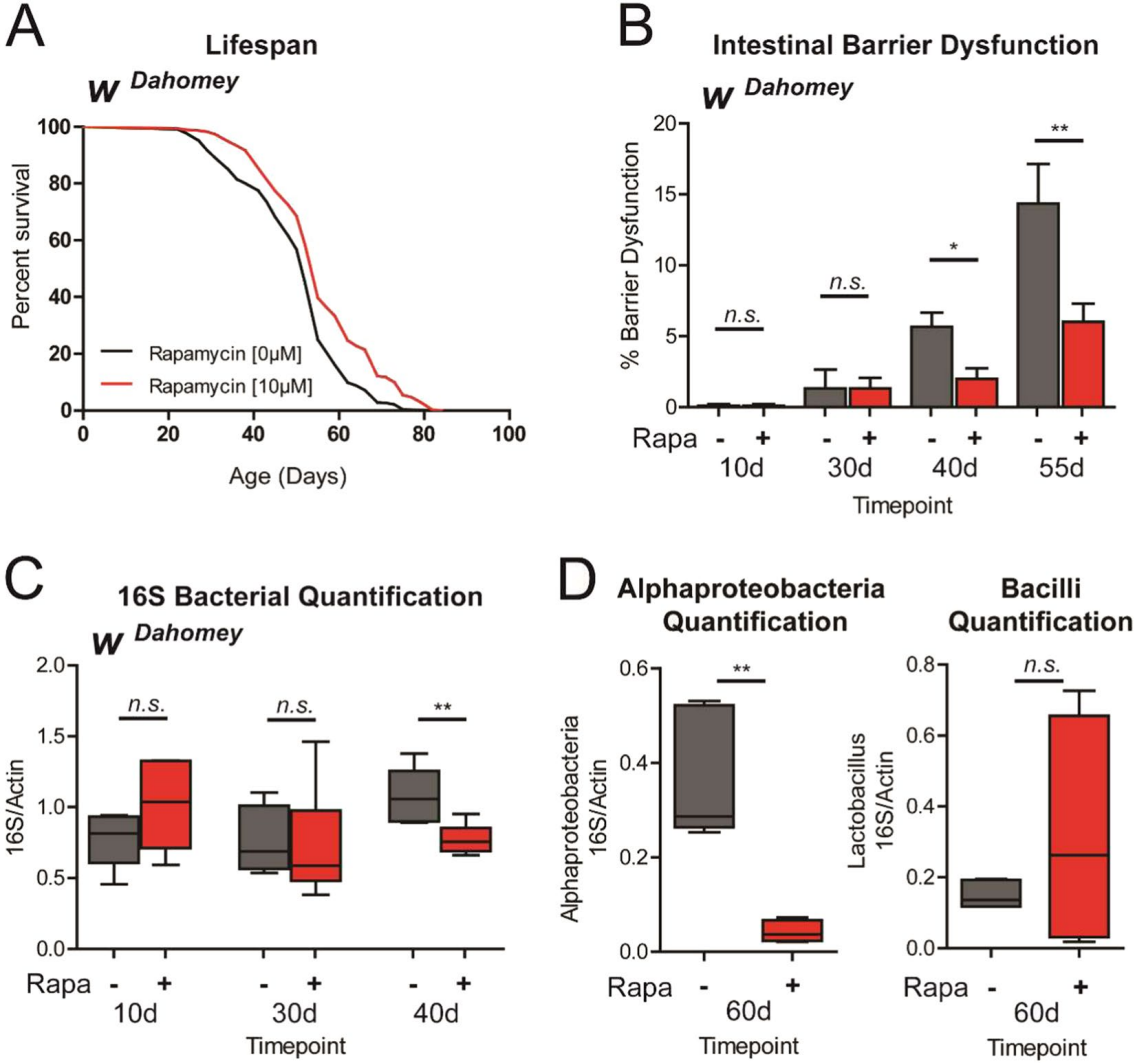
Rapamycin treatment improves gut health and modulates microbiota composition during aging. (**A**) Survival curves of *w*^*Dahomey*^ females with or without rapamycin treatment from day 4 onwards (post-eclosion). p < 0.0001, log rank test; n > 225 flies. Representative result of 4 separate lifespan trials. (**B**) Intestinal integrity during aging of *w*^*Dahomey*^ females with or without rapamycin treatment from day 4 onwards (post-eclosion). *p < 0.05, **p < 0.01, one-way ANOVA/Bonferroni’s multiple comparisons test; n = 300 flies on day 10 per treatment. (**C**) Bacterial levels assayed by qPCR of the 16S rRNA gene in surface sterilized, non-smurf *w*^*Dahomey*^ females with or without rapamycin treatment from day 4 onwards (post-eclosion). **p < 0.01, Mann-Whitney U-test; n = 6 replicates of five flies per timepoint. (**D**) Bacterial levels assayed by taxon specific primers in surface sterilized, non-smurf *w*^*Dahomey*^ females fed with or without rapamycin from day 4 onwards. **p < 0.01, Mann-Whitney U-test; n = 6 replicates of five flies per timepoint. Rapamycin was provided in the media at a concentration of 10 μg/ml for (**A**–**D**). All error bars represent SEM.

The fruit fly *Drosophila melanogaster* is an excellent model organism to explore the interplay between microbiota composition, intestinal health and aging^21,34^. In the first place, we confirmed that treating adult female flies with rapamycin supplemented in their food can prolong fly lifespan. Consistent with previous findings^8^, we observed that rapamycin-treated flies display a significant increase in both mean and maximum lifespan in multiple trials (Figs 1A and S1A). Intestinal barrier dysfunction is a common feature of aging organisms and has been linked to a number of human diseases^35–37^. Indeed, loss of intestinal barrier function has been reported in aged worms^38,39^, flies^38,40,41^, fish^38^, rodents^28^ and monkeys^42^. Importantly, in *Drosophila* at least, intestinal barrier dysfunction is a harbinger of age-onset mortality^23,41^. Hence, we assayed whether rapamycin could delay the onset of intestinal barrier dysfunction in aging flies. To do so, we utilized the Smurf assay to examine intestinal integrity during aging^40,41^. Consistent with a previous report^32^, we found that rapamycin treatment during adulthood delays the onset of intestinal barrier dysfunction (Figs 1B and S1B). A number of studies have demonstrated that microbial load in the *Drosophila* intestine increases with age^22,23,43–45^. It has been reported that rapamycin treatment reduces the number of colony-forming units (CFUs) in dissected guts from aged flies^32^. Since flies that display intestinal barrier dysfunction (Smurf flies) show increased microbial loads^23^, it is possible that the reported reduction in CFUs is linked to reduced numbers of Smurf flies. To better understand the impact on commensal homeostasis during aging, we utilized qPCR with universal primers to the bacterial 16S rRNA gene to characterize alterations in microbiota dynamics in response to rapamycin treatment in non-Smurf flies. Rapamycin treatment led to a significant reduction in bacterial loads (Figs 1C and S1C), with these changes occurring before detectable intestinal barrier dysfunction (in non-Smurf flies). Recent work has shown that increased microbial loads are also associated with a shift in bacterial composition toward a greater proportion of Proteobacteria species in aged flies and that this shift is more closely associated with the health status of the animal than with chronological age^23^. Hence, we next utilized primers to the 16S rRNA gene that are specific to the classes Bacilli and Alphaproteobacteria^23^. Upon rapamycin treatment, there was a decrease in Alphaproteobacteria but no significant change in Bacilli (Figs 1D and S1D,E), with this change also occurring in non-Smurf flies. Together, these findings show that rapamycin treatment limits age-related microbial dysbiosis and improves gut health in aged flies.

### Rapamycin requires autophagy to improve gut health and maintain muscle proteostasis in aged flies

**Figure 2:**
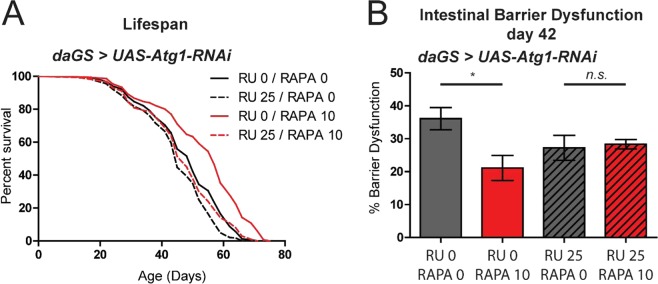
Rapamycin treatment requires autophagy to improve gut health during aging. (**A**) Survival curves of *daGS* > *UAS-Atg1-RNAi* female flies treated with (red) or without rapamycin (black), and with (dashed lines) or without RU486 (solid lines) from day 4 onwards. p < 0.0001, log rank test; n > 205 flies. Representative result of 2 separate lifespan trials shown. (**B**) Intestinal integrity of day 42 *daGS* > *UAS-Atg1-RNAi* female flies treated with (red) or without (black) rapamycin, and with (dashed lines) or without (solid lines) RU486 from day 4 onwards. *p < 0.05, one-way ANOVA/Bonferroni’s multiple comparisons test; n = 180 flies per treatment. Rapamycin was provided in the media at a concentration of 10 μg/ml and RU486 at a concentration of 25 μg/ml for (**A**,**B**). All error bars represent SEM.

Macroautophagy, hereafter autophagy, is a degradation pathway in which cellular materials are sequestered by double-membrane vesicles known as autophagosomes, and delivered to the lysosome for degradation^46^. Autophagy has been shown to play important roles in aging and lifespan determination in diverse species^47^. More specifically, autophagy induction has been implicated as a causal factor in numerous prolongevity interventions^47^. Indeed, it has been reported that ubiquitous knockdown of *Atg5*, a gene required for autophagy, during development and adulthood prevents rapamycin-mediated lifespan extension in flies^8^. Here, we set out to build upon this finding by determining whether rapamycin-mediated longevity requires autophagy specifically in adult flies. To this end, we utilized the RU486-inducible, Gene-Switch expression system^48^ to induce adult-onset knock-down of *Atg1*, which is involved in the initiation of autophagosome formation^18,49^. We used the ubiquitous *daughterless* (*da*)GS driver line to inhibit *Atg1* by RNAi in adult flies (Fig. S2A) and compared survivorship with and without rapamycin treatment. Adult-onset RNAi of *Atg1* completely suppressed rapamycin-mediated longevity (Figs 2A and S2B,C). Next, we set out to determine whether the rapamycin-mediated beneficial effects on intestinal barrier function in aging flies requires functional autophagy. Critically, the ability of rapamycin to improve intestinal integrity in aged flies is dependent upon *Atg1* gene activity. Feeding rapamycin to control flies improved intestinal barrier function at day 42 (Figs 2B and S2D). However, adult-onset RNAi of *Atg1* prevented this rapamycin-mediated improvement in gut health (Figs 2B and S2D). Together, these findings support the idea that rapamycin requires a functional autophagy pathway, in adults, to promote gut health and longevity.

**Figure 3:**
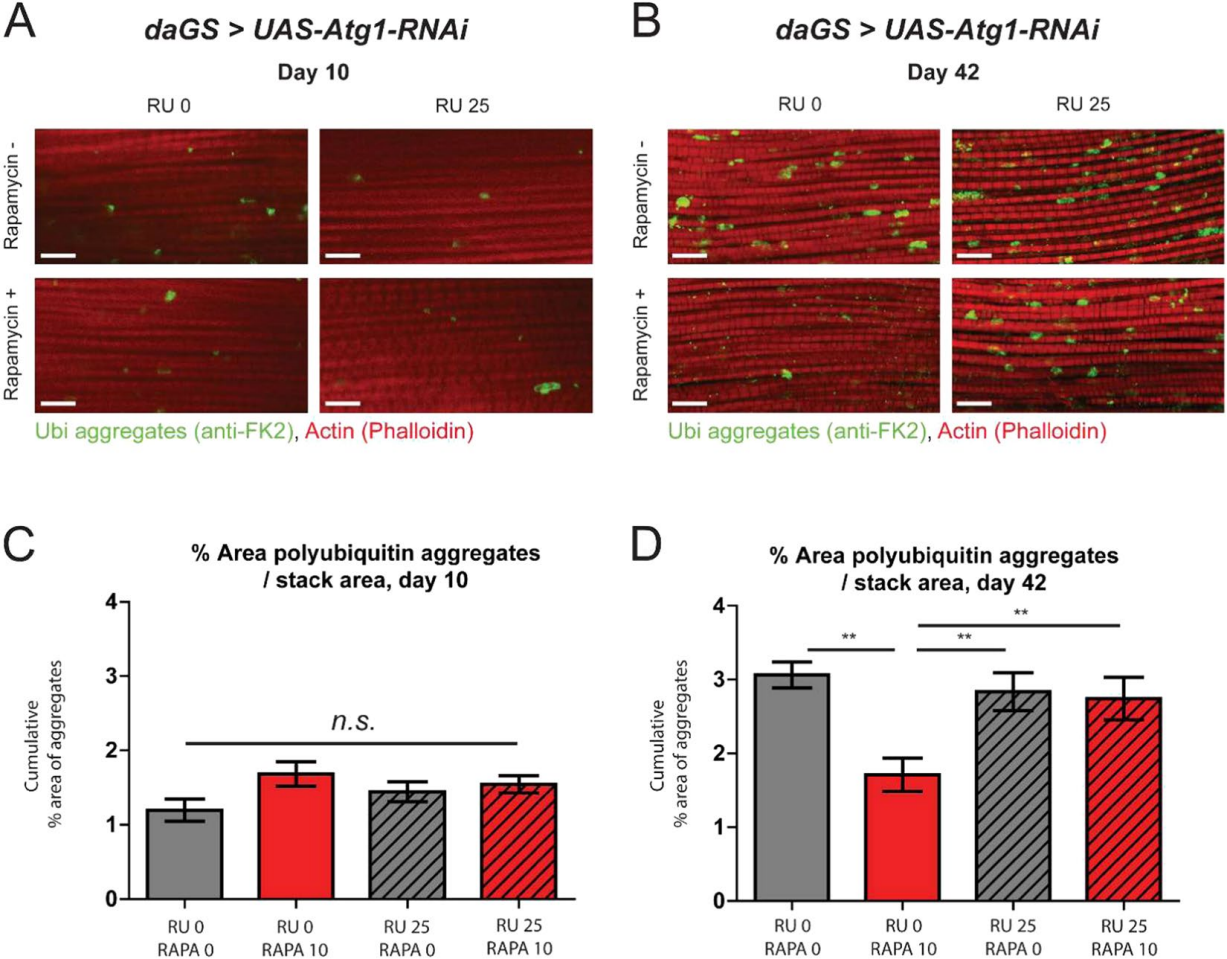
Rapamycin treatment maintains proteostasis during aging in an autophagy-dependent manner. (**A**,**B**) Immunostaining of indirect flight muscles from 10 and 42 day old *daGS* > *UAS-Atg1-RNAi* female flies with or without both rapamycin and RU486 treatment from day 4 onwards (post-eclosion), showing polyubiquitinated aggregates (green channel, anti-FK2) and muscles (red channel stained with phalloidin/F-Actin). Scale bar is 10 µm. (**C**,**D**) Quantifications of polyubiquitin aggregates in muscle as shown in (**A**,**B**) respectively. **p < 0.01, one-way ANOVA w/ Newman-Keuls post-test; n > 12, one fly per replicate. Rapamycin was provided in the media at a concentration of 10 μg/ml and RU486 at a concentration of 25 μg/ml for (**A**–**D**). All error bars represent SEM.

The age-related loss of muscle function and mass, sarcopenia, is a key feature of health decline in the elderly^50^. Studies in model organisms have revealed that loss of proteostasis is a major contributing factor to muscle aging^51^. Indeed, muscle aging in *Drosophila* is correlated with an accumulation of ubiquitinylated, insoluble protein aggregates^52^. We sought to determine if rapamycin was capable of improving proteostasis during muscle aging. To this end, we characterized the accumulation of ubiquitinated protein aggregates in aged flight muscles using immunofluorescence microscopy. As previously reported^52–55^, we observed that aged *Drosophila* flight muscles accumulate ubiquitinated protein aggregates consistent with a loss of proteostasis (Fig. 3A,B). Feeding rapamycin had no impact on the levels of ubiquitinated protein aggregates in young flies (Fig. 3A,C). Importantly, however, feeding rapamycin from early adulthood lead to a significant reduction in ubiquitinated protein aggregates in aged muscle (Fig. 3B,D). Autophagy has been shown to be involved in maintaining muscle proteostasis in aging organisms^47,52^. Hence, we examined whether the rapamycin-mediated improvement in proteostasis in aged flies requires autophagy. We used the ubiquitous *da*GS driver line to inhibit *Atg1* by RNAi in adult flies and compared the accumulation of protein aggregates with and without rapamycin treatment. Adult-onset RNAi of *Atg1* completely suppressed rapamycin-mediated improvements in proteostasis in aged muscle (Fig. 3B,D). Hence, the ability of rapamycin to delay this marker of muscle aging is dependent upon *Atg1* gene activity.

### Rapamycin can extend lifespan and healthspan in the absence of a microbiota

**Figure 4:**
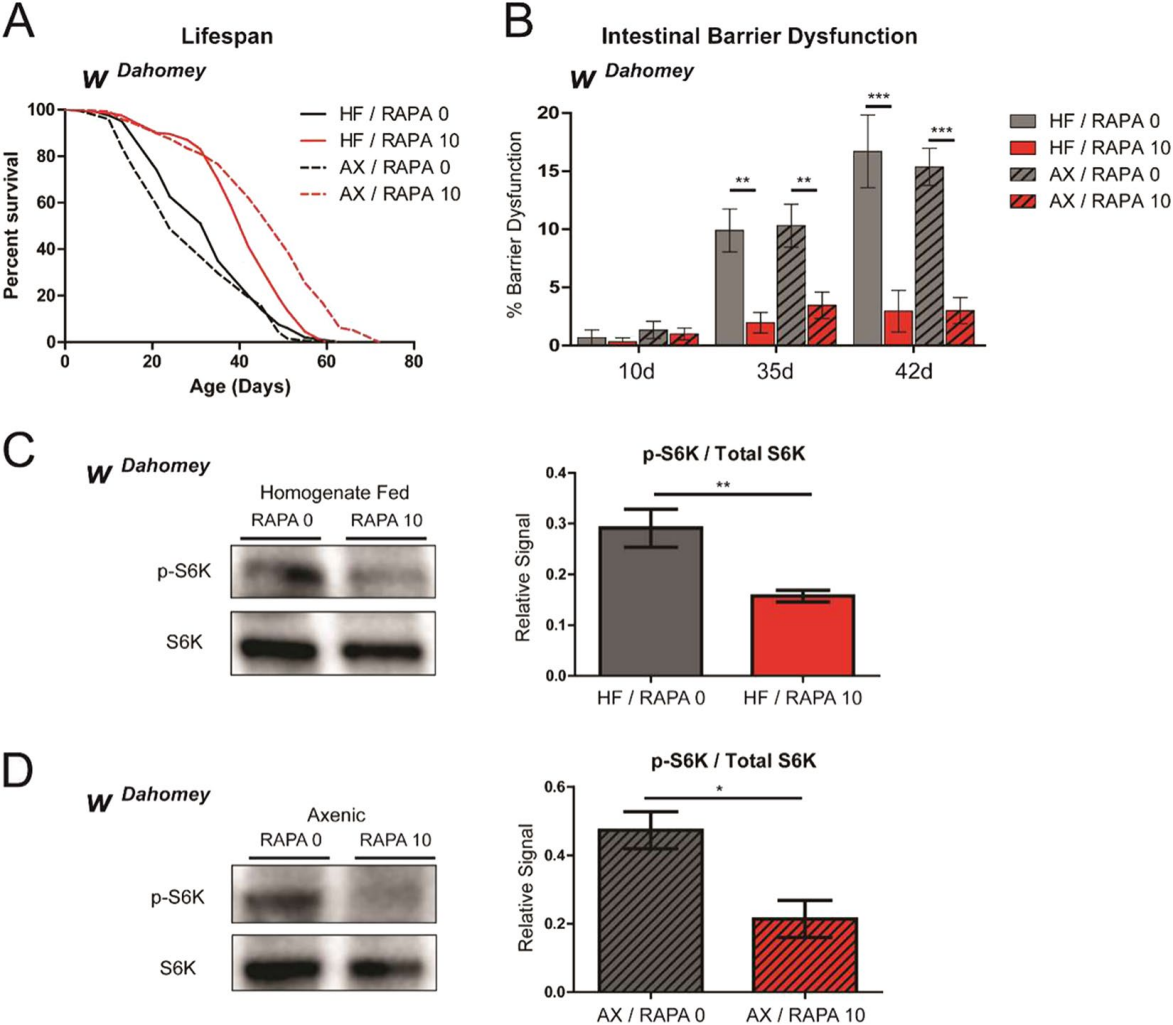
Rapamycin treatment extends lifespan, improves barrier function and reduces TOR signaling in germ-free flies. (**A**) Survival curves of *w*^*Dahomey*^ female flies rendered germ free as embryos, and either maintained germ free (AX, dashed lines) or refed with a bacterial homogenate (HF, solid lines) and treated with (red) or without (black) rapamycin from day 4 onwards (post-eclosion). ***p < 0.0001, log rank test; n ≥ 200 flies. Representative result of 3 separate lifespan trials shown. (**B**) Intestinal integrity of day 10, 35 and 42 *w*^*Dahomey*^ flies with (HF, solid bars) or without homogenate feeding (AX, dashed bars) and with (red) or without rapamycin (black) treatment from day 4 onwards (post-eclosion). **p < 0.01 and ***p < 0.001, one-way ANOVA/Bonferroni’s multiple comparisons test; n = 300 flies at day 10 per treatment. (**C**) Western blot detection and densitometry of p-S6K (T398) and total S6K levels from day 10 homogenate fed (HF) *w*^*Dahomey*^ flies treated with or without rapamycin from day 4 onwards. n = 5 replicates, 10 flies per replicate; **p < 0.01; Mann-Whitney U-test. (**D**) Western blot detection and densitometry of p-S6K (T398) and total S6K levels from day 10 axenic (AX) *w*^*Dahomey*^ flies treated with or without rapamycin from day 4 onwards. n = 5 replicates, 10 flies per replicate; *p < 0.05; Mann-Whitney U-test. Rapamycin was provided in the media at a concentration of 10 μg/ml. All error bars represent SEM.

Having ascertained that alterations in microbiota dynamics are correlated with improved lifespan and tissue homeostasis upon rapamycin treatment, we next asked whether these effects were in any way microbiota-dependent. To this end, we generated cohorts of flies rendered germ-free as described previously^23^, and either maintained axenically or reintroduced to fly homogenate. We then examined the impact of rapamycin treatment on lifespan under both germ-free and control (homogenate-fed) conditions. Critically, we found that rapamycin was capable of extending lifespan in both the homogenate-fed and axenic conditions in multiple trials (Figs 4A and S3A), and, thus, this phenotype was not dependent upon the microbiota. Age-related microbial dysbiosis contributes to intestinal barrier dysfunction in aged animals^21,23,28^. As rapamycin treatment limits age-onset dysbiosis (Fig. 1C,D), we tested whether rapamycin-mediated improvements in intestinal integrity are dependent upon the microbiota. However, we found that rapamycin was able to improve intestinal barrier function during aging in both the homogenate fed and axenic conditions (Fig. 4B). Next, we examined the ability of rapamycin treatment to inhibit TOR activity *in vivo* in axenic and control flies. To do so, we examined phosphorylation of S6K, a well-described downstream target of TORC1^5^, as an indicator of TOR activity. We confirmed that TOR signaling was significantly reduced by rapamycin treatment in microbe-bearing control flies, as measured by the ratios of phosphorylated S6K relative to total S6K (Figs 4C and S3C). Furthermore, we found that rapamycin treatment significantly reduced TOR signaling in axenic flies, suggesting that the efficacy of rapamycin is not dependent upon the microbiota (Figs 4D and S3C).

To further examine the role of rapamycin-mediated changes on the microbiota on aging, we extracted a bacterial homogenate from aged flies fed rapamycin, and from age-matched controls. These ‘rapamycin-fed’ and control homogenates were then refed to young axenic flies. Interestingly, however, no change in lifespan was found between these two cohorts (Fig. S3B), despite causing persistent changes in bacterial load and composition (Fig. S3D,E). Together, our findings indicate that the effects of rapamycin treatment on the microbiota are not necessary or sufficient to promote longevity.

### The efficacy of rapamycin treatment on proteostasis is enhanced in germ-free flies

**Figure 5:**
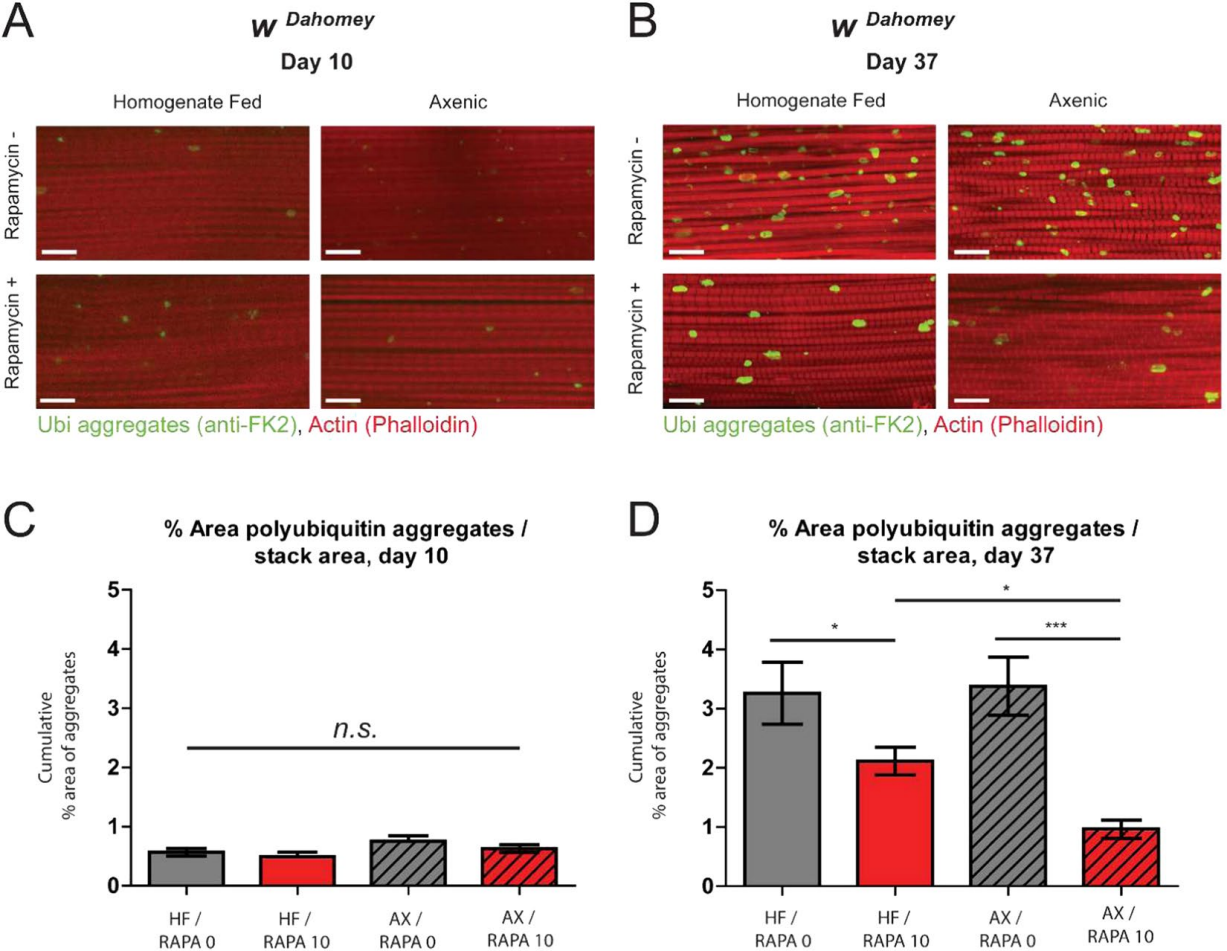
The efficacy of rapamycin treatment on proteostasis is enhanced in the absence of a microbiota. (**A**,**B**) Immunostaining of indirect flight muscles from 10 and 37 day old w^*Dahomey*^ female flies rendered germ free as embryos, and either maintained germ free (AX) or refed a bacterial homogenate (HF), and treated with or without rapamycin from day 4 onwards, showing polyubiquitinated aggregates (green channel, anti-FK2) and muscles (red channel stained with phalloidin/F-Actin). Scale bar is 10 µm. (**C**,**D**) Quantifications of polyubiquitin aggregates in muscle as shown in (**A**,**B**) respectively. *p < 0.05, ***p < 0.001, one-way ANOVA w/ Newman-Keuls post-test; n > 13, one fly per replicate. Rapamycin was provided in the media at a concentration of 10 μg/ml for (**A**–**D**). All error bars represent SEM.

When comparing the prolongevity effects of rapamycin treatment on germ-free and control flies (Figs 4A and S3A), we noted that the magnitude of lifespan extension was more pronounced in germ-free flies. To better understand this observation, we examined the interplay between rapamycin-mediated improvements in proteostasis and the microbiota. More specifically, we examined rapamycin-mediated alterations in the accumulation of ubiquitinated protein aggregates in aged flight muscles in control and axenic flies (Fig. 5A–D). Feeding rapamycin had no impact on the levels of ubiquitinated protein aggregates in young flies (Fig. 5A,C). Importantly, we observed a significant reduction in the levels of ubiquitinated protein aggregates in axenic flies treated with rapamycin compared to microbe-bearing controls (Fig. 5B,D). Taken together, our results suggest that a microbiota is neither necessary nor sufficient to confer the beneficial effects of rapamycin at the cellular, tissue or organismal level, and in some cases is indeed more efficacious in the absence of a microbiota.

## Discussion

Interventions that target the underlying aging process have the potential to transform human health. A better understanding of whether prolongevity interventions are influenced by or dependent upon microbiota composition may be critical in assessing whether these approaches are likely to prove effective in promoting healthy aging in a broad spectrum of aged individuals. Rapamycin has emerged as a leading candidate to target aging and forestall disease, extending lifespan and healthspan in a variety of organisms. It has also been reported previously that in addition to improving lifespan, rapamycin treatment induces significant changes to the host microbiota, lowering bacterial load in flies and modulating the microbiome in mammals^33^. We report similar changes in the *w*^*Dahomey*^ strain of *Drosophila* under our laboratory settings. To assess the relevance of these changes to the prolongevity effects of rapamycin, we report the first examination of the impact of rapamycin treatment on aging in germ-free animals. We observed that removal of the microbiota did not prevent the ability of rapamycin to prolong lifespan, revealing that rapamycin’s effects on the microbiota are not necessary for lifespan extension in this context. Conversely, we found that reassociation of germ-free flies with the microbiota of rapamycin-treated conventional flies had no effect on host lifespan, suggesting rapamycin-induced microbiota changes are insufficient in themselves to convey prolongevity effects. One of the physiological hallmarks of aging that we assayed was loss of intestinal barrier function. In previous work, we have shown that different strains of flies display different rates of age-onset intestinal barrier dysfunction^41^. Although the underlying mechanisms are not fully understood, it is plausible that genetic differences between strains and an interplay with microbiota composition result in alterations in gut homeostasis during aging. In any case, here, we report that rapamycin can delay the onset of intestinal barrier dysfunction in axenic flies. Moreover, we found the magnitude of lifespan extension observed upon rapamycin treatment was enhanced in germ-free flies compared to controls, and that rapamycin treatment leads to a greater improvement in proteostasis in germ-free flies, suggesting the presence of a microbiota may even slightly hamper the beneficial effects of rapamycin treatment. Recent studies have shown an association between microbe abundance and lifespan extension or other metabolic readouts, which is modulated by diet^56–59^. Taken together, these findings suggest that the ability of rapamycin to mediate lifespan extension in axenic flies might also be affected by nutrition. It is important to note that our experiments were carried out on a single diet.

The importance of interactions between the microbiota and therapeutic treatments has been highlighted by recent work demonstrating a role for the intestinal microbiota in determining patient responses to a particular cancer therapy^60–62^. These interactions may take place in either direction; the impact of a given therapeutic on the microbiota may be required for its action in the host, or the microbiota may act upon the therapeutic in ways that modify its function. A number of drugs are known to be metabolized by the microbiota^63^, with either beneficial or detrimental effects on drug function. In addition, it has been reported that microbes can bolster or suppress the effects of fluoropyrimidines, used to treat colorectal cancers, in *C*. *elegans*^64^. In our experimental setting we find that the action of rapamycin on the microbiota is not required for its beneficial effects. However, our observation that the presence of a microbiota may reduce the efficacy of rapamycin treatment suggests that further investigation of the interaction between rapamycin and the microbiota is warranted. Indeed, it is possible that our findings may help explain published reports that fail to observe rapamycin-mediated lifespan extension in flies^65^. In conclusion, our study confirms that feeding rapamycin can impact both lifespan, and microbiota dynamics during aging. However, in our experimental setting, we have found that rapamycin’s effect on the microbiota is neither necessary nor sufficient to bring about significant lifespan gains in the host. It is of great interest whether or not our observations will be replicated in other model systems, or if they are unique to *Drosophila*.

## Materials and Methods

### Fly culture and media

The majority of this work was carried out in the standard laboratory strain *w*^*Dahomey*^. Additional genotypes used were the *da*-GS GeneSwitch line received from H. Tricoire (Université Paris Diderot–Paris 7), and the *UAS-Atg1-RNAi* line, received from the Vienna Drosophila RNAi (VDRC) Center (stock no. 16133). Flies were cultured in a humidified, temperature-controlled incubator with 12 h on/off light cycle at 25 °C, in vials containing standard cornmeal medium (1% agar, 3% brewer’s yeast, 1.9% sucrose, 3.8% dextrose and 9.1% cornmeal; all concentrations given in wt/vol). Rapamycin (LC Labs) and RU486 (Cayman Chemical Company) were dissolved in ethanol and mixed into the media when preparing food vials. The rapamycin dose used was 10 ug/ml, and the RU486 dose was 25 ug/ml. Control food had the same total volume of ethanol vehicle (1/400, 100% ethanol) as experimental food.

### Lifespan analysis

Flies that eclosed over a 36-hour period were collected and allowed to mate for approximately 60 hours. Female or male flies were collected under light nitrogen-induced anesthesia and maintained at a density of 30+/−3 flies per vial in a humidified, temperature-controlled (25 °C) incubator with a 12 hour light/dark cycle. Flies were transferred to new vials every 2–3 days and scored for the number of deaths per vial.

### Intestinal barrier dysfunction assay

The ‘Smurf’/intestinal barrier dysfunction assay was performed similarly to^41^. Flies were aged on standard medium until the day of the Smurf assay. Dyed medium was prepared using standard medium with blue dye no. 1 (SPS Alfachem) added at a concentration of (2.5% wt/vol). Flies were then allowed to consume dyed food for 24 hours prior to quantification. A fly was counted as a Smurf when dye coloration was observed outside the digestive tract.

### Genomic DNA isolation

Genomic DNA was extracted using the PowerSoil DNA isolation kit (MoBio). All flies were surface sterilized as previously described^22^ prior to sample preparation. To ensure consistent homogenization, whole fly samples were pre-homogenized in 150 µL of solution from the PowerSoil bead tube using a motor pestle. This homogenate was then returned to the bead tube and the manufacturers protocol was followed.

### Quantitative PCR

DNA samples for qPCR of the 16S ribosomal RNA gene were prepared as described above. RNA extractions, for analysis of the knockdown of the Atg1 gene, were carried out in TRIzol (Invitrogen) as per the manufacturer’s directions. cDNA synthesis was carried out using the First Strand cDNA Synthesis Kit (Fermentas). PCR was performed with *PowerUp* SYBR Green Master Mix (Applied Biosystems) on a CFX96 Real Time PCR system (BioRad). Cycling conditions were as follows: 95 °C for10 minutes; 95 °C for 15 s then 60 °C for 60 s, cycled 40 times. All calculated gene expression values were normalized to the value of the loading control gene Actin5C. The following primer sequences were used:

Act5C_L –TTGTCTGGGCAAGAGGATCAG, Act5C_R - ACCACTCGCACTTGCACTTTC;

Atg1_F- GCTTCTTTGTTCACCGCTTC, Atg1_R- GCTTGACCAGCTTCAGTTCC.

Universal primers for the 16 S ribosomal RNA gene were against variable regions 1 (V1F) and 2 (V2R), as previously published^66^. Taxon specific 16S primers were as follows:

Bacilli_F – CGACCTGAGAGGGTAATCGGC, Bacilli_R – GTAGTTAGCCGTGGCTTTCTGG;

Alpha_F – CCAGGGCTTGAATGTAGAGGC, Alpha_R – CCTTGCGGTTCGCTCACCGGC

### Immunostaining procedure for dissected indirect flight muscle

Hemithoraces were dissected and fixed for 20 minutes in PBS with 4% paraformaldehyde and 0.2% Triton X-100. After washing, samples were incubated overnight at 4 °C with an antibody detecting polyubiquitinated proteins at 1:250 mouse mAb FK2 (Enzo). Washed thoroughly and incubated with secondary anti-mouse AlexaFlour-568 (1:250), with phalloidin AlexaFluor-633. Samples were rinsed three times in PBS + 0.2% triton X-100 for 10 minutes at room temperature, then mounted in Vectashield mounting medium (Vector Labs) and imaged. For quantification of protein aggregates in hemithoraces, single-channel images were converted into grayscale and the area and total number and % area of protein aggregates was measured using ImageJ.

### Generation of axenic and re-associated flies

To generate axenic (germ-free) flies, embryos were treated by bleach and ethanol as described previously^67^. Briefly, <12-h-old embryos were dechorionated in 3% sodium hypochlorite (50% v/v regular bleach) for 20 min, rinsed in 70% ethanol for 5 min, and then washed three times with 1 × PBS + 0.01% Triton X-100. Axenic embryos were transferred to autoclaved media vials in a laminar flow cabinet. Axenic conditions within each vial were confirmed by plating swabs from spent vials on MRS agar before flies were taken as samples or pooled into a lifespan, as per^68^. To generate flies associated with microbes as embryos, whole fly homogenate (10 fly equivalent: 600 μL of conventionally reared fly homogenate glycerol stock/bottle) was added to medium containing axenic embryos.

### Preparation of fly homogenate for re-association and adult feeding

Conventionally reared adult flies were surface sterilized by 70% ethanol prior to homogenization to ensure only internal microbes were present in the homogenate. Surface sterile flies were homogenized with a motor pestle in 1.5 mL tube with 200 μL of sterile PBS (50 flies/tube). Homogenates were then pooled and sterile PBS added to adjust to one fly equivalent in 50 μL PBS. For storage 1/5 volume of 80% sterile glycerol was added and aliquots were stored at −80 °C until use. For adult feeding, freshly prepared homogenate (one fly equivalent in 50 μL PBS) was added to standard food vials and allowed to dry. Unless otherwise noted, homogenate from 10 day post-eclosion *w*^*Dahomey*^ flies was used for homogenate-fed controls. To test the effects of rapamycin-induced changes to the microbiota on host lifespan, homogenate was prepared from 42 day post-eclosion *w*^*Dahomey*^ females reared either on rapamycin media or ethanol control media as outlined above.

### Western blot

Samples for western blot were obtained from 10 day old female flies (head and thorax only). Samples were homogenized and lysates were separated by SDS page using standard procedures. Membranes were probed with primary antibodies against anti-phospho-p70 S6K T398 (dilution 1:2000, Cell Signaling, 9209) and anti-total S6K 1:300 (dilution 1:500, Santa Cruz, C-18, SC-230). The rabbit antibodies were detected using horseradish peroxidase-conjugated anti-rabbit IgG antibodies at 1:2000 (Sigma) and the mouse antibodies were detected using horseradish peroxidase-conjugated anti-mouse IgG antibodies 1:2000 dilution (Sigma). Amersham ECL chemiluminescent/chemifluorescent reagent (GE Healthcare) was used to visualize horse radish peroxidase activity, and the chemifluorescence was detected using a PXi Touch scanner (Syngene).

### Statistics

All statistical tests were implemented in GraphPad Prism. The comparison of survival curves was done using the log-rank test. Comparison of Smurf proportion per time point was carried out using one-way ANOVA with Bonferroni’s multiple comparison’s test. Comparisons of ubiquitin aggregates were tested for significant differences using one-way ANOVA with the Newman-Keuls post-test. All other data comparisons were tested for significant differences using the Wilcoxon-Mann-Whitney U-test. All statistical tests are two-sided.

## Supplementary information

Schinaman et al Supp. Data File

## Data Availability

The datasets generated during and/or analyzed during the current study are available from the corresponding author on reasonable request.
