# Supplementary material for: Rapamycin modulates tissue aging and lifespan independently of the gut microbiota in Drosophila
Source: Sci Rep. 2019 May 24;9:7824. doi: 10.1038/s41598-019-44106-5 (PMC6534571; doi:10.1038/s41598-019-44106-5)
Supplement: Supplementary file 1 — Schinaman et al Supp. Data File [file 41598_2019_44106_MOESM1_ESM.docx]

**Supplementary Information:**

Rapamycin modulates tissue aging and lifespan independently of the gut microbiota in *Drosophila*

Joseph M. Schinaman^1^, Anil Rana^1^, William W. Ja^2^, Rebecca I. Clark^3^ & David W. Walker^1,4*^

1. Department of Integrative Biology and Physiology, University of California, Los Angeles, Los Angeles, California 90095, USA.
2. Department of Neuroscience, The Scripps Research Institute, Jupiter, FL 33458, USA; Center on Aging, The Scripps Research Institute, Jupiter, FL 33458, USA
3. Department of Biosciences, Durham University, Durham DH1 3LE, UK
4. Molecular Biology Institute, University of California, Los Angeles, Los Angeles, California 90095, USA.

* Correspondence:

David W. Walker, Ph.D.

Email: [davidwalker@ucla.edu](mailto:benzer@caltech.edu)


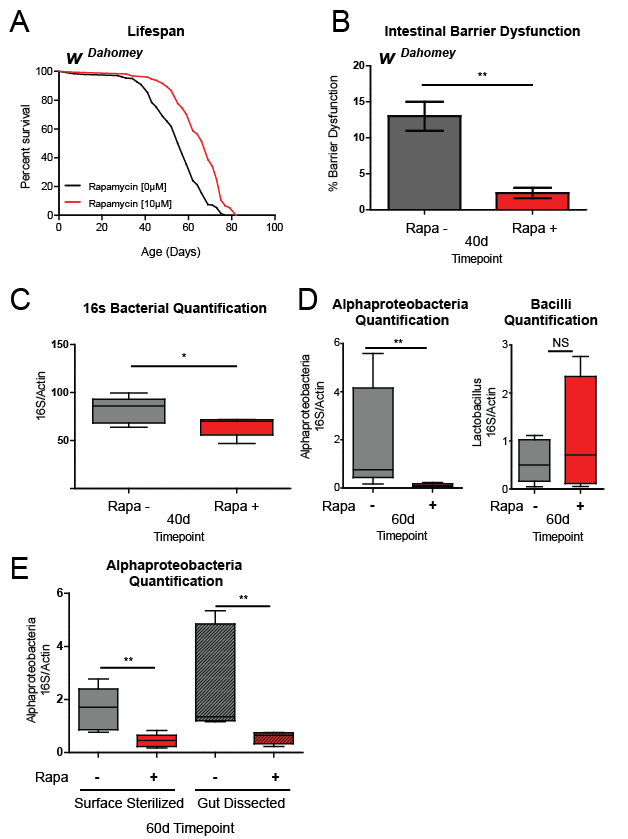
**­­­­**

**Figure S1, related to Figure 1. Effect of rapamycin on lifespan, intestinal integrity and microbiota.**

(**A**) Repeat of lifespan assay of w*^Dahomey^* female flies treated with 10 uM rapamycin (red) or without rapamycin (black) from 4 days post eclosion (p < 0.0001; log rank test; n > 225 flies). (**B**) Repeat of smurf assay of w*^Dahomey^* females fed rapamycin (red) or not (grey) at the day 40 timepoint (p < 0.01 at 40 days post eclosion, Mann-Whitney U-test). (**C**) Repeat of whole bacterial level assessment by qPCR of the 16S rRNA gene in surface sterilized, non-smurf w*^Dahomey^* females fed rapamycin (red) or not (grey) at the day 40 timepoint (p < 0.05, Mann-Whitney U-test, n= 6 replicates of five flies per timepoint). (**D**) Repeat of bacterial level assessment by taxon specific primers in surface sterilized, non-smurf w*^Dahomey^* females fed rapamycin (red) or not (grey) late in life (p < .01, Mann-Whitney U-test, n = 6 replicates of five flies per timepoint). (E) Comparison of taxon specific bacterial levels in surface sterilized flies (solid boxes) vs. dissected guts (dashed boxes) of non-smurf w*^Dahomey^* females fed rapamycin (red) or not (grey) at the late life timepoint (p < .01, Mann-Whitney U-test, n = 6 replicates of five flies for timepoint for surface sterilized flies, n = 5 replicates for gut dissections). Similar levels of significance are

**
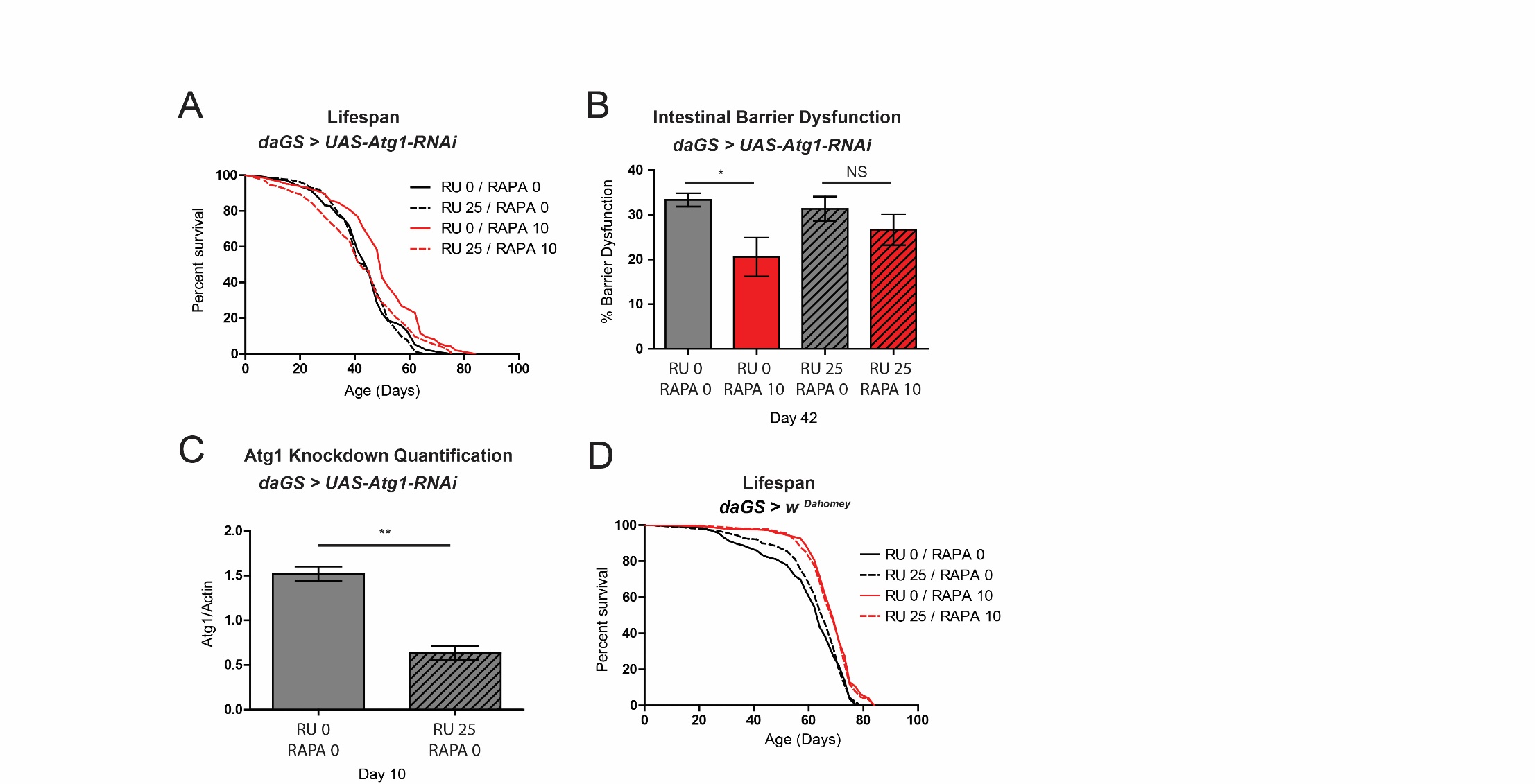
**

**Figure S2, related to Figure 2- The effect of rapamycin on lifespan and barrier function requires a functioning autophagy pathway.**

(**A**) Repeat of lifespan assay of *da-GS>UAS-Atg1-RNAi* female flies treated with rapamycin (red) or not (black), and with RU486 (dashed lines) or without RU486 (solid lines) (p < 0.0001; log rank test; n > 280 flies). (**B**) Repeat of smurf assay of *da-GS>UAS-Atg1-RNAi* female flies treated with rapamycin (red) or not (black), and with RU486 (dashed lines) or without RU486 (solid lines) (p < 0.05; one-way ANOVA/Bonferroni’s multiple comparisons test, 42 days post eclosion). (**C**) Quantitative PCR analysis of Atg1 transcript levels of *da-GS>UAS-Atg1-RNAi* female flies treated with RU486 (red) or without RU486 (grey) (p < 0.01; Mann-Whitney U-test , n = 6 replicates of six flies each). (**D**) Lifespan assay of *da-GS>w^Dahomey^* female flies treated with rapamycin (red) or not (black), and with RU486 (dashed lines) or without RU486 (solid lines) (p < 0.0001; log rank test; n > 240 flies).

**­­**
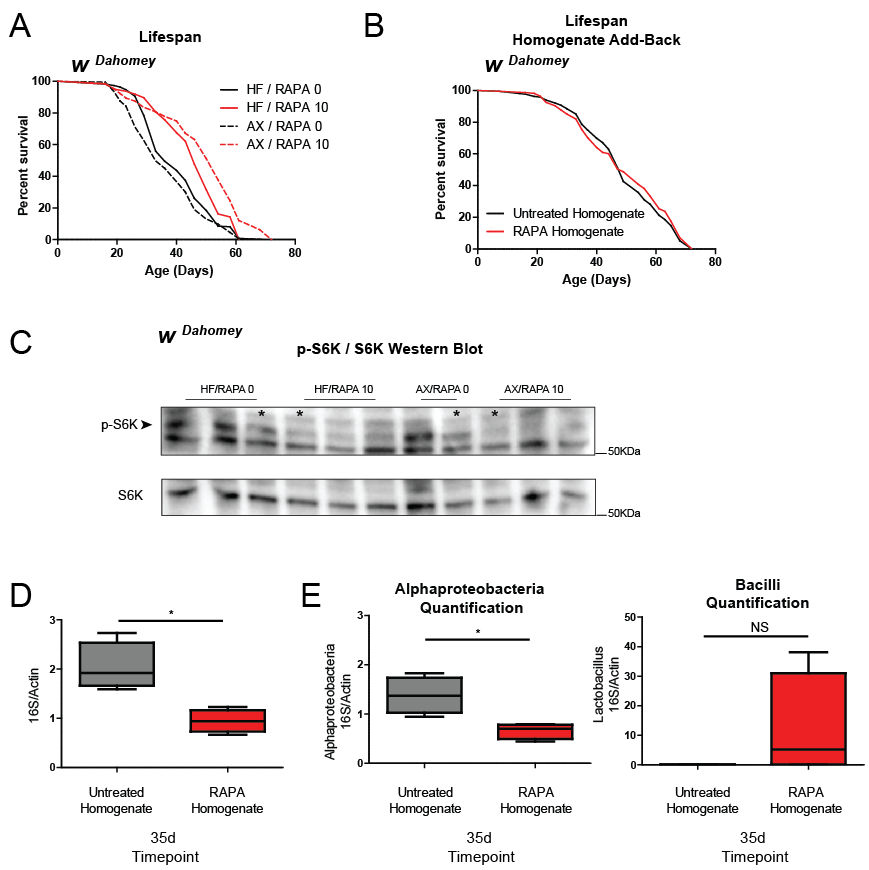


**Figure S3, related to Figure 4-**

**(**A) Repeat of lifespan assay of w*^Dahomey^* female flies rendered germ free as embryos, and either maintained germ free (AX) or refed a bacterial homogenate (HF), and treated with either rapamycin (red) or not (black) (p < 0.0001; log rank test; n > 200 flies). (**B**) Lifespan assay of w*^Dahomey^* female flies rendered germ free as embryos, and inoculated once at age 4 days post-eclosion with a bacterial homogenate from 42-day-old flies treated either with rapamycin (red) or ethanol control (black). No effect was found between the two homogenates (p > 0.05, log rank test, n > 215 flies). (**C**) Complete western blot gel image showing p-S6K (T398) and total S6K levels from day 10 homogenate fed (HF) and axenic (AX) *w^Dahomey^* flies treated with or without rapamycin from day 4 onwards. Asterisks in the image indicate representative lanes shown in Fig. 4C and 4D. (**D**) Whole bacterial level assessment by qPCR of the 16S rRNA gene in surface sterilized, non-smurf w*^Dahomey^* females fed homogenates as described in (B) at the day 35 timepoint ( p < 0.05, Mann-Whitney U-test, n= 4 replicates of five flies per timepoint). (**E**) Bacterial level assessment by taxon specific primers in surface sterilized, non-smurf w*^Dahomey^* females fed homogenates as described in (B) at day 35 timepoint (p < .05, Mann-Whitney U-test, n = 4 replicates of five flies per timepoint).
